# The heat shock protein 70 gene as a new alternative molecular marker for the taxonomic identification of *Streptomyces* strains

**DOI:** 10.1186/s13568-018-0674-4

**Published:** 2018-09-10

**Authors:** Yang Hu, Fengxian Sun, Weiying Liu

**Affiliations:** 1 Department of Pathogen Biology, School of Basic Medical Sciences, Tianjin Medical University, 22 Qi-Xiang-Tai Road, Tianjin, 300070 China; 2 Department of Physiology and Pathophysiology, School of Basic Medical Sciences, Tianjin Medical University, Tianjin, 300070 China

**Keywords:** 16S rRNA, Molecular marker, *hsp70*, *gyrB*, Taxonomic identification

## Abstract

With the developments in taxonomy, the classically used highly conserved 16S rRNA molecular marker has shown some disadvantages among closely related species. For further taxonomic studies of the prokaryotes, specific PCR primers were designed from two conserved regions in the amino acid sequences of the 70-kDa heat shock protein sourced from 20 different genera in actinomycetes. These were used for the amplification of the *hsp*70 genes in 16 *Streptomyces* strains. Then, we investigated the phylogenetic relationships among these *Streptomyces* strains and compared the tree topology based on the *hsp70* gene with those based on the previously used markers (16S rRNA and *gyrB*). To our knowledge, this is the first use of the *hsp*70 gene as a molecular marker for the taxonomic identification of *Streptomyces.*

## Introduction

The highly conserved 16S rRNA gene, also known as the ‘bacterial fossil’, encodes small subunit of the ribosomal RNA (rRNA) in prokaryotes. It contains 10 variable regions and 11 constant regions (Woese et al. 1975). Phylogenetic analysis based on the 16S rRNA gene is considered to be a simple and appropriate tool for the construction of bacterial phylogenetic relationships. Thus, it is one of the most commonly used methods for identifying microorganisms (Amann et al. 1995; Stackebrandt and Goebel 1994; Woese et al. 1990). Recently, molecular techniques based on the PCR, such as DGGE, LH-PCR, cPCR, SSCP, ARDRA, AFLP, RFLP, FISH and T-RFLP, have provided outstanding tools for the detection, identification, and characterisation of microorganisms based on this gene region (Giraffa and Neviani 2001). However, the high percentage of sequence similarity among closely related species limits its effectiveness (Ash et al. 1991; Christensen et al. 1998; Yamamoto and Harayama 1998).

Consequently, other protein-encoding gene sequences have been evaluated for use as tools in phylogenetic and taxonomic identification (Küpfer et al. 2006). The *gap* and *omp*A genes, which encode glyceraldehyde-3-phosphate dehydrogenase and outer-membrane protein 3A respectively, were used to identify five species of *Escherichia*, but these did not form a monophyletic group (Lawrence et al. 1991). In addition, the sequences of *rec*A, *rpo*B, *inf*B, *fus*A and *nif*D, which are involved in DNA recombination or DNA repair, the RNA polymerase β-subunit and translation initiation factor 2, the protein synthesis elongation factor-G and the α subunit of the dinitrogenase protein were also used to describe the phylogenetic relationships of different bacterial genera (Das et al. 2014; Hedegaard et al. 1999; Holmes et al. 2004; Lloyd and Sharp 1993; Mollet et al. 1997). Unfortunately, despite having a higher phylogenetic resolution than the 16S rRNA sequences (Yamamoto and Harayama 1996), some of the phylogenetic trees did not form distinct phylogenetic groups (Dauga 2002). Recently, the *gyr*B gene, which encodes the subunit B protein of DNA gyrase, was proposed as a suitable phylogenetic marker. It was most commonly used in the identification and classification of the evolutionary relationships of closely related species, due to its success (Kasai et al. 2000; Venkateswaran et al. 1998; Yamada et al. 1999; Yamamoto et al. 1999; Yamamoto and Harayama 1998).

Heat shock proteins (HSPs) are groups of stress-response proteins that have been demonstrated to be universally distributed across biological groups from bacterial to human beings (Hunt and Morimoto 1985). According to their biological activities and apparent molecular weights, HSPs are classified into four major families: *hsp*90, *hsp*70, *hsp*60, and small HSPs including *hsp*27. A study previously revealed that human *hsp*70 is 73% identical to *Drosophila hsp*70 and is 47% identical to *Escherichia coli Dna*K. These have been considered as useful phylogenetic markers (Hunt and Morimoto 1985). We aimed to develop a set of novel PCR primers that allow for the amplification of the *hsp*70 gene. These were then used to study the phylogenetic relationships among *Streptomyces* strains.

## Materials and methods

### Strains and culture conditions

All the *Streptomyces* strains used in this study were collected in our laboratory. *E. coli* strains were typically cultured in Luria–Bertani (LB) medium or on LB agar plates at 37 °C (Sambrook and Russell 2001). Where appropriate, the media contained the antibiotic ampicillin (1 μg/mL). To prepare the total genomic DNA of *Streptomyces*, Tryptone Soy Broth liquid medium containing 0.5% glycine was used for culturing the strains (Hopwood et al. 1985).

### Chemicals and reagents

All the chemicals and reagents used were of the highest purity commercially available. Taq polymerase was purchased from TaKaRa Bio Inc. (Japan).

### Preparation of genomic DNA

All bacteria genomic DNA was isolated using the method described by Hopwood (Hopwood et al. 1985).

### PCR amplification of target genes

PCR amplification was performed using an Eppendorf Personal Master Cycler. A master mix of 50 μL used for the PCR reaction contained: 5.0 μL 10X PCR Buffer, 3.0 μL MgCl_2_ (25 mM), 4.0 μL dNTPs (2.5 mM each), 1.0 μL of degenerate PCR primers (20 mM each), 2.5 μL dimethyl sulfoxide, 1.0 μL genomic DNA (ca.0.2 μg) and 0.5 μL Taq polymerase. The amplification conditions were as follows: (a) 1 cycle for 3 min at 94 °C; (b) 94 °C for 1 min, 64 °C for 1 min, then 72 °C for 1.5 min for 35 cycles; (c) 1 cycle at 72 °C for 10 min.

In addition, the 16S rRNA gene and the *gyrB* gene were amplified by PCR using the primers as described previously (Calcutt 1994; Edwards et al. 1989). The PCR products were directly sequenced by an ABI Genetic Analyzer 3730 (Invitrogen Bio Inc., Shanghai, China) which used the 16S rRNA primers and F-1S (5′-GAGGTCGTGCTGACCGTGCTGCA-3′)/F-2R (5′-GTTGAT GTGCTGGCCGTCGACGT-3′) for *gyr*B (Hatano et al. 2003).

### Gel analysis and DNA sequencing

All PCR products were analyzed using 1.0% agarose-gel electrophoresis, stained with ethidium bromide (AMRESCO, USA). PCR products were directly sequenced by ABI Genetic Analyzer 3730 (Invitrogen Bio Inc., Shanghai, China) which used the sequence primers U1F (5′-CGTGCAGTCGGTATCGACCTCGG-3′) and 2R (5′-CGATGCCGTTGGCGTCGATGTC-3′).

### Phylogenetic analysis

16S rRNA and *hsp*70 gene sequences were compared to the GenBank nucleotide and protein databases using BLASTN and BLASTX (http://blast.ncbi.nlm.nih.gov/Blast.cgi). Phylogenetic and molecular evolutionary analyses were conducted using MEGA 4.1 (Tamura et al. 2007). Briefly, for the construction of bootstrap test of 16S rRNA and *hsp* 70 gene phylogenetic trees, the algorithm for the neighbour-joining (NJ) method (Saitou and Nei 1987) and Kimura 2-parameter model (Kimura 1980) were used. Percentage bootstrap values based on 1000 resampled datasets are shown.

#### Nucleotide sequence accession numbers

All the sequences used in this paper were deposited into GenBank under the accession numbers JF443561–JF443576 and HQ607409–HQ607473, as indicated in Fig. 3.

## Results

### Design primers for the PCR amplification of *hsp*70 genes

**Fig. 1:**
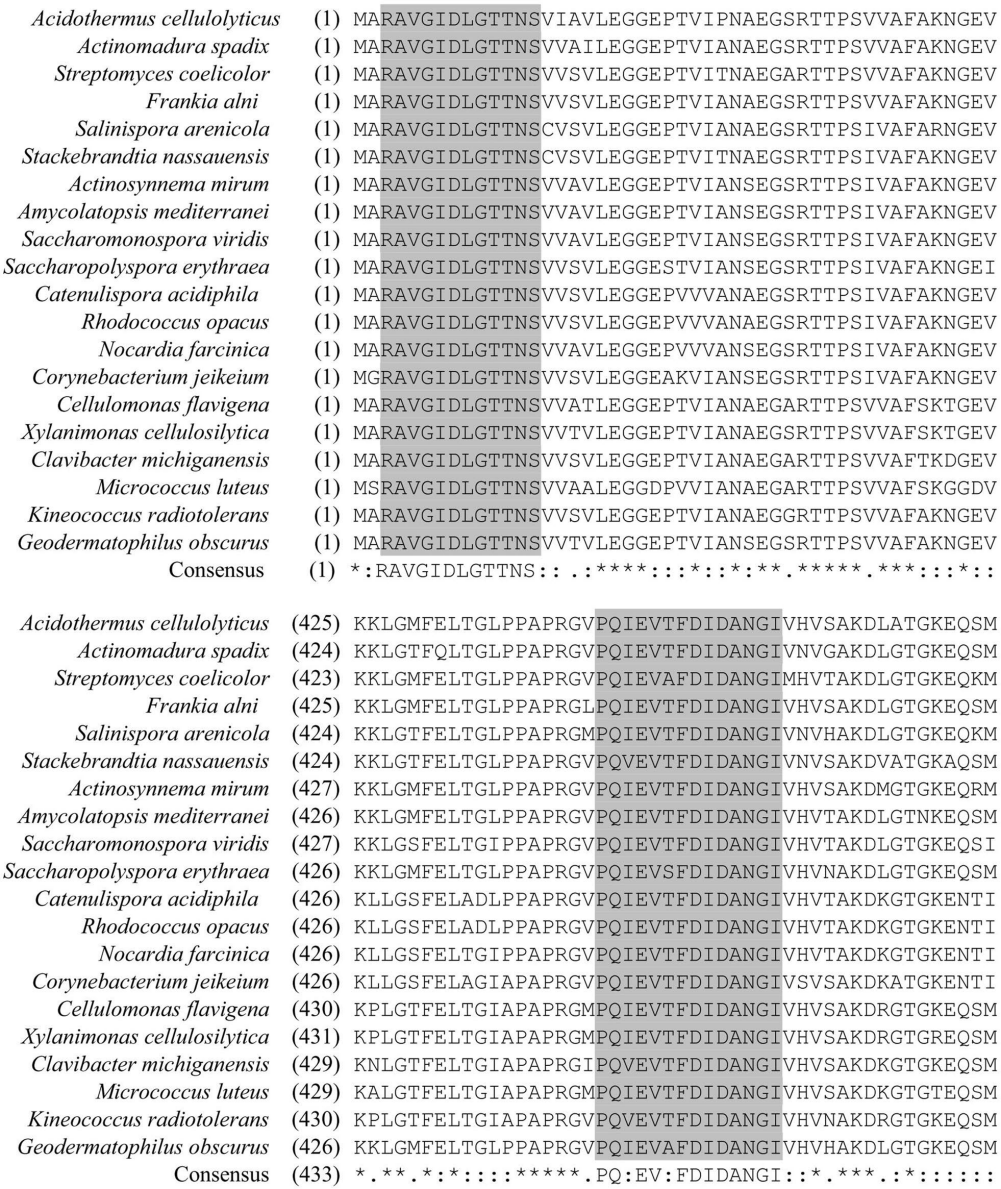
Sequence alignment of approximately 480N-terminal amino acids of 20 selected 70-kDa heat shock proteins from actinomycetes. Each protein sequences were deposited in GeneBank (*Acidothermus. cellulolyticus*: CP000481; *Actinomadura spadix*: AF451887; *Streptomyces coelicolor*: L46700; *Frankia alni*: CT573213; *Salinispora arenicola*: CP000850; *Stackebrandtia. nassauensis*: CP001778; *Actinosynnema mirum*: NC_013093; *Amycolatopsis mediterranei*: CP002000; *Saccharomonospora viridis*: CP001683; *Saccharopolyspora erythraea*: AM420293; *Catenulispora acidiphila*: CP000431; *Rhodococcus opacus*: AP011115; *Nocardia farcinica*: AP006618; *Corynebacterium jeikeium*: CR931997; *Cellulomonas flavigena*: CP001964; *Xylanimonas cellulosilytica*: CP001821; *Clavibacter michiganensis*: AM849034; *Micrococcus luteus*: CP001628; *Kineococcus radiotolerans*: CP000750; *Geodermatophilus obscurus*: CP001867). “*” indicates where the residues are identical in all sequences in the alignment in that column. “:”, “.” indicates where conserved and semi-conserved substitutions are observed, respectively. Two conserved regions of the amino acid sequences enclosed in grey are used to design degenerated primers for the amplification of approximately 1300 bp *hsp*70 gene fragment

PCR primers for the amplification of *hsp*70 (*Dna* K) genes were designed from two conserved regions of the amino acid sequences of the 70-kDa heat shock protein from 20 genera in the actinomycetes (Fig. 1). Then, one primer pair was designed for amplifying approximately 1300 bp of the *hsp*70 gene fragment and the other primer pair was used to direct sequencing (Table 1). The primer pairs were aligned with the other gene sequences in the GenBank using BLAST (http://www.ncbi.nlm.nih.gov/BLAST).

**Table 1:** Primers used for the PCR amplification of the *hsp*70 gene of *Streptomyces* strains

Primer	Oligonucleotide sequence^a^	Length (bases)	Positions^b^	Orientation
*hsp*70-U7F	CGTGCAGTCGGTATCGACCTCGGBACVACBAACTC	35	7–41	Sense
*hsp*70-1326R	CGATGCCGTTGGCGTCGATGTCGAASGHSACCTCGA	36	1326–1363	Antisense
U1F^c^	CGTGCAGTCGGTATCGACCTCGG	23	7–29	Sense
2R^c^	CGATGCCGTTGGCGTCGATGTC	22	1326–1347	Antisense

^a^IUB code for degenerated base positions: B = G/C/T; V = A/G/C; S = C/G; H = A/C/T

^b^Positions correspond to the *hsp*70(*Dna*K) nucleotide sequence of *S. coelicolor* (GeneBank NO. L46700)

^c^Direct sequencing primers for *hsp*70-U7F/*hsp*70-1326R

### PCR amplification of *hsp*70 genes

**Fig. 2:**
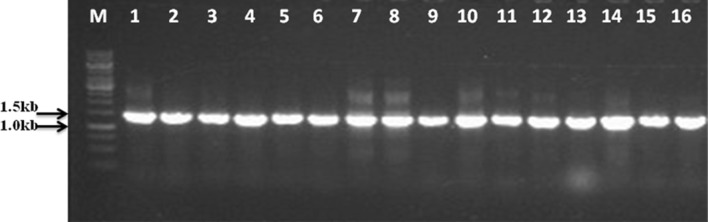
Gel electrophoresis of PCR products amplified with degenerate primers (*hsp*70-U7F and *hsp*70-1326R). Lanes M, 1 kb DNA marker; lanes 1 to 16, *S. tendae* strain 944, *S. pluricolorescens* strain 999, 1043 *S. panayensis* strain 1043, *S. flavovariabilis* strain 1067, *S. macrosporeus* strain 1076, *S. drozdowiczii* strain 1136, *S. olivaceus* strain 1137, *S. chartreusis* strain 1159, *S. malaysiensis* strain 1161, *S. werraensis* strain 1165, *S. albogriseolus* strain 1168, *S. hawaiiensis* strain 1183, *S. castaneoglobisporus* strain 1185, *S. phaeofaciens* strain 1187, *S. acrimycini* strain 1202

Primer optimization experiments were performed using a single primer pair (*hsp*70-U7F and *hsp*70-1326R) to amplify the *hsp*70 gene of *Streptomyces coelicolor*. The results showed that the sequences of the PCR product amplified from the primer pair was the same as the *hsp70* genes retrieved from the Genbank database. Then, the primer pair described above was used to amplify the *hsp*70 genes in 16 *Streptomyces* strains. The PCR products with the expected size of approximately 1300 bp corresponded to those predicted from known *hsp*70 sequences (Fig. 2).

### Comparison of *hsp*70 and 16S rRNA phylogenetic tree topologies

**Fig. 3:**
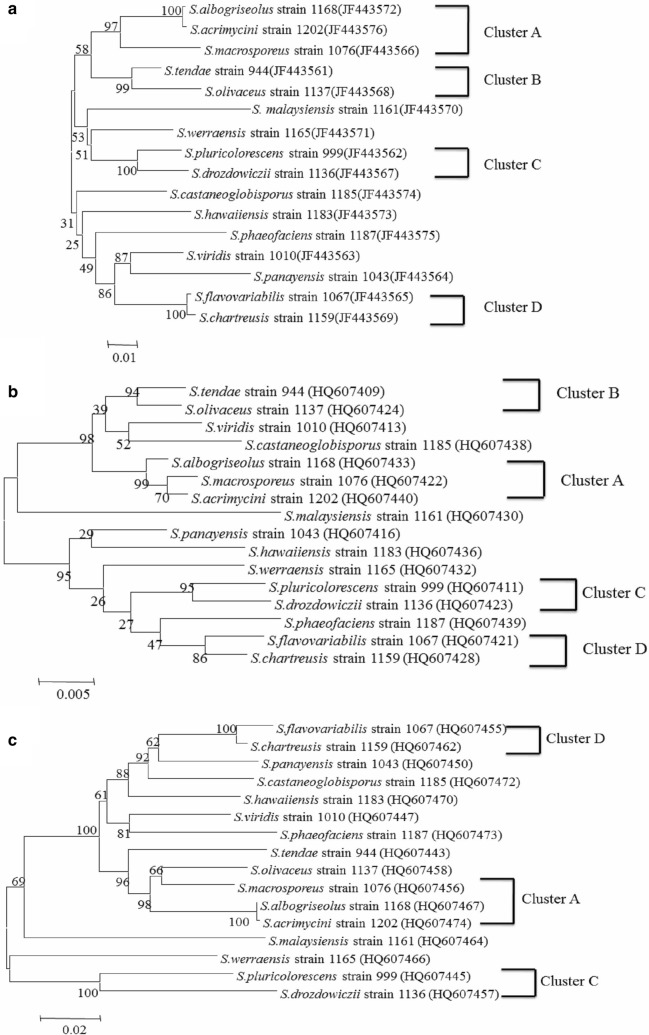
Phylogenetic relationships based on the *hsp70* (**a**), 16S rRNA (**b**) and *gyrB* (**c**) gene sequences of 16 *Streptomyces* strains

When the phylogenetic trees constructed from the nucleotide sequences of *hsp70* and the 16S rRNA gene from the same 16 S*treptomyces* reference strains were compared, analysis showed there were only slight differences between the phylogenetic structures for these *Streptomyces*. Based on the bootstrap values and the level of the stability of the branches, the 16 strains of *Streptomyces* were divided into four stable subgroups (A, B, C and D) (Fig. 3a, b). According to the *hsp70* phylogenetic tree, two strains, *S. albogriseolus* strain 1168 and *S. acrimycini* strain 1202, clustered closely in the A subgroup branch with a bootstrap value of 100%, whereas *S. acrimycini* strain 1202 and *S. macrosporeus* strain 1076 clustered closely in the 16S rRNA phylogenetic tree with a bootstrap value of 70%. Therefore, subgroup A of *hsp70* phylogenetic tree showed higher stability than the 16S rRNA phylogenetic tree. Furthermore, the strains in B, C and D subgroups also displayed higher stability for the *hsp70* phylogenetic tree compared to the phylogenetic tree constructed from 16S rRNA. In addition, two strains, *S. viridis* strain 1010 and *S. panayensis* strain 1043, were outside the four subgroups and clustered closely in *hsp70* phylogenetic tree with a bootstrap value of 87%, and *S. viridis* strain 1010 clustered closely with *S. castaneoglobisporus* strain 1185; *S. panayensis* strain 1043 clustered closely with *S. hawaiiensis* strain 1183 with bootstrap values of 52% and 29%, respectively. These results showed that the phylogenetic tree constructed from *hsp70* had a reasonable topology in comparison to the 16S rRNA phylogenetic tree.

### Comparison of *hsp*70 and *gyrB* phylogenetic tree topologies

Comparison between the *hsp70* and *gyrB* phylogenetic trees showed that there were some inconsistencies, leaving only 3 subgroups A, C and D (Fig. 3a, c). In both phylogenetic trees, these 3 subgroups were congruent and stable, but *hsp70* phylogenetic tree was even more reasonable. For example, in the *hsp70* and *gyrB* phylogenetic trees, subgroup D clustered closely with *S. panayensis* strain 1043 with a bootstrap value 87% and 62%, respectively; it seems that the branch in the *hsp70* phylogenetic tree had a reasonable topology. Also, in the *hsp70* phylogenetic tree, *S. viridis* strain 1010 and *S. panayensis* strain 1043 formed a more stable branch, but there were some differences from the phylogenetic tree based on *gyrB*. Among the phylogenetic trees (*hsp70*, 16S rRNA and *gyrB*), it seems that the phylogenetic relationships of *S. viridis* strain 1010 and *S. panayensis* strain 1043 in the *hsp70* phylogenetic tree was more reasonable.

## Discussion

In modern taxonomy classifying bacteria, the 16S rRNA and *gyrB* gene sequences have been considered to be simple and the most commonly used methods for the investigation of phylogenetic relationships (Dauga 2002; Fox et al. 1980; Fukushima et al. 2002; Hillis and Dixon 1991). Previous research has shown that the average substitution rate for 16S rRNA was 1% per 50 million years which is slower than that of *gyrB* gene (0.7%–0.8% per million years) (Ochman and Wilson 1987). Thus, the *gyrB* sequence analysis may better distinguish the phylogenetic relationships at the species level in some bacterial groups. However, this phylogenetic approach also has its limitations. Recently, *rpoB* and *recA* sequence analysis along with randomly amplified polymorphic fingerprinting could effectively identify *Bacillus* species with highly similar 16S rRNA gene sequences; these were hard to identify based on 16S rDNA sequence analysis (Mohkam et al. 2016). Further, several mass spectrometry methods were used for the classification and identification of bacteria and other microorganisms (Sauer and Kliem 2010). For example, MALDI-TOF MS analysis was demonstrated to be a rapid method for bacteria identification from mining samples (Avanzi et al. 2017; Książczyk et al. 2016; Schröttner et al. 2016). Although many new analytical tools were recently used for the classification and identification of bacteria, there is still urgency for the discovery of new molecular markers.

The 60 kDa heat-shock protein family *hsp65* gene has also been used successfully for species identification of cultured clinical isolates of the genus *Mycobacterium* (Rogall et al. 1990). The *hsp70* gene which is highly conserved between 16S rRNA and *gyrB* sequences is distributed universally from bacteria to human beings (Hunt and Morimoto 1985). Consequently, it has the potential to be used as a phylogenetic marker for the investigation of phylogenetic relationships. However, using *hsp70* gene as a molecular marker for taxonomic identification is still rare to our knowledge.

Therefore, in this study, we attempted to develop novel PCR primers (*hsp*70-U7F and *hsp*70-1326R) for the investigation of the phylogenetic relationships of *Streptomyces* strains through amplification of the *hsp*70 gene. Furthermore, the PCR products can be directly and rapidly sequenced by the sequence primers U1F and 2R. Compared with the phylogenetic tree based on the 16S rRNA gene and the *gyrB* gene, the *hsp70* phylogenetic tree has advantages for the investigation of the phylogenetic relationships of *Streptomyces* strains. Firstly, analysis based on the *hsp70* provided a resolving power higher than that of the 16S rRNA gene analysis in differentiating among species. Secondly, separation among closely related species such as *S. panayensis* strain 1043, *S. viridis* strain 1010 and *S. phaeofaciens* strain 1187, show that the *hsp70* phylogenetic tree provided convincing as well as reasonable phylogenetic relationships.

In conclusion, for the first time, this study develops novel PCR primers (*hsp*70-U7F and *hsp*70-1326R) were used for the investigation of the phylogenetic relationships between 16 *Streptomyces* strains through amplification of *hsp*70 gene. This gene may be a good alternative as a molecular marker for the phylogenetic analysis of *Streptomyces* strains. In the future, *hsp*70 combined with 16S rRNA and *gyrB* sequence method may be able to clarify molecular relationship of some controversial phylogenetic relationships of bacteria.

## Abbreviations

hsp70: heat shock protein 70
gyrB: DNA gyrase, subunit B
PCR: polymerase chain reaction
DGGE: denaturing gradient gel electrophoresis
LH-PCR: length heterogeneity polymerase chain reaction
cPCR: comparative polymerase chain reaction
SSCP: single stranded conformation polymorphisms
ARDRA: amplified ribosomal DNA restriction analysis
AFLP: amplified fragment length polymorphism
RFLP: restriction fragment length polymorphism
FISH: fluorescence in situ hybridization
T-RFLP: terminal restriction fragment length polymorphisms
